# Genetic Deletion of NOD1 Prevents Cardiac Ca^2+^ Mishandling Induced by Experimental Chronic Kidney Disease

**DOI:** 10.3390/ijms21228868

**Published:** 2020-11-23

**Authors:** Marta Gil-Fernández, José Alberto Navarro-García, Almudena Val-Blasco, Laura González-Lafuente, José Carlos Martínez, Angélica Rueda, Maria Tamayo, José Luis Morgado, Carlos Zaragoza, Luis Miguel Ruilope, Carmen Delgado, Gema Ruiz-Hurtado, María Fernández-Velasco

**Affiliations:** 1 IdiPAZ: Hospital La Paz Institute for Health Research, 28046 Madrid, Spain; martagf1995@gmail.com (M.G.-F.); almudena.valblasco@gmail.com (A.V.-B.); 2 Cardiorenal Translational Laboratory, Institute of Research i+12, Hospital Universitario 12 de Octubre, 28041 Madrid, Spain; Jalbertong@gmail.com (J.A.N.-G.); laura.gonzlafuente@gmail.com (L.G.-L.); marsanjosec@gmail.com (J.C.M.); ruilope@icloud.com (L.M.R.); 3 Departamento de Bioquímica, Centro de Investigación y de Estudios Avanzados del IPN, México City 07360, Mexico; arueda@cinvestav.mx; 4 Biomedical Research Institute “Alberto Sols” CSIC-UAM, 28046 Madrid, Spain; mtamayo@iib.uam.es (M.T.); biomorgui@hotmail.com (J.L.M.); cdelgado@iib.uam.es (C.D.); 5 Departamento de Cardiología, Unidad de Investigación Mixta Universidad Francisco de Vitoria, 28223 Madrid, Spain; c.zaragoza.prof@ufv.es; 6 Centro de Investigación Biomédica en Red en Enfermedades Cardiovasculares (CIBERCV), 28029 Madrid, Spain; 7 School of Doctoral Studies and Research, European University of Madrid, 28224 Madrid, Spain; 8 CIBER-CV, Hospital Universitario 12 de Octubre, 28029 Madrid, Spain

**Keywords:** NOD1, chronic kidney disease, RIP2, RyR_2_, Ca^2+^ handling

## Abstract

Risk of cardiovascular disease (CVD) increases considerably as renal function declines in chronic kidney disease (CKD). Nucleotide-binding oligomerization domain-containing protein 1 (NOD1) has emerged as a novel innate immune receptor involved in both CVD and CKD. Following activation, NOD1 undergoes a conformational change that allows the activation of the receptor-interacting serine/threonine protein kinase 2 (RIP2), promoting an inflammatory response. We evaluated whether the genetic deficiency of *Nod1* or *Rip2* in mice could prevent cardiac Ca^2+^ mishandling induced by sixth nephrectomy (Nx), a model of CKD. We examined intracellular Ca^2+^ dynamics in cardiomyocytes from *Wild-type* (*Wt*), *Nod1*^−/−^ and *Rip2^−/−^* sham-operated or nephrectomized mice. Compared with *Wt* cardiomyocytes, *Wt*-Nx cells showed an impairment in the properties and kinetics of the intracellular Ca^2+^ transients, a reduction in both cell shortening and sarcoplasmic reticulum Ca^2+^ load, together with an increase in diastolic Ca^2+^ leak. Cardiomyocytes from *Nod1*^−/−^-Nx and *Rip2*^−/−^-Nx mice showed a significant amelioration in Ca^2+^ mishandling without modifying the kidney impairment induced by Nx. In conclusion, *Nod1* and *Rip2* deficiency prevents the intracellular Ca^2+^ mishandling induced by experimental CKD, unveiling new innate immune targets for the development of innovative therapeutic strategies to reduce cardiac complications in patients with CKD.

## 1. Introduction

Chronic kidney disease (CKD) is a complex pathology characterized by a reduced glomerular filtration rate, increased urinary albumin excretion and kidney damage [1]. Recent studies have found that cardiac complications are frequent in patients with CKD [2,3]. The United States Renal Data System (USRDS) 2014 annual report stated that the prevalence of any cardiovascular disease (CVD) is about 2-fold higher in patients with CKD than in patients without CKD. Heart failure (HF) is the main cardiovascular risk in patients with CKD from the outset, and increases gradually with the progression of renal dysfunction [4]. Patients with CKD also have a higher prevalence of cardiac systolic and diastolic dysfunction [5]. These data underscore the sharp increase in mortality in advanced CKD, due mainly to the occurrence of cardiovascular events, such as arrhythmias [6,7]. The complex association between CKD and cardiac dysfunction can likely be explained by the clustering of several risk factors, including uremia and inflammatory mediators [8,9]. Yet, little is known about the underlying mechanisms of cardiac dysfunction in CKD. 

Cardiac muscle cell contraction is tightly regulated by the change in intracellular Ca^2+^ levels, acting as a key mediator of excitation-contraction (EC)-coupling. The initial depolarization event of the action potential activates L-type Ca^2+^ channels (LTCCs) of the sarcolemma, firing an inward voltage-dependent Ca^2+^ current type l (I_CaL_) from the extracellular medium. Ca^2+^ entry triggers a large release of Ca^2+^ from the sarcoplasmic reticulum (SR) by ryanodine receptor (RyR_2_) channels, resulting in an increased intracellular Ca^2+^ concentration ([Ca^2+^]_i_) that prompts cell contraction. For relaxation to occur, [Ca^2+^]_i_ must return to diastolic levels and this occurs mainly by two mechanisms: (i) Ca^2+^ re-uptake by the Sarco/Endoplasmic Ca^2+^ pump (SERCA) 2a and (ii) Ca^2+^ extrusion by the Na^+^/Ca^2+^ exchanger (NCX). During diastole, RyR_2_ channels are mostly closed; however, there is always a low but finite probability that a RyR_2_ channel will spontaneously open, mediating Ca^2+^ flux into the cytosol—known as Ca^2+^ sparks. Ca^2+^ spark frequency is normally low during diastole but, in some pathological conditions, abnormally large or frequent sparks can activate the RyR_2_ channels at neighboring release sites, generating SR Ca^2+^ waves that favor Ca^2+^ extrusion by NCX, and providing a substrate to initiate a life-threatening arrhythmia. Dysregulation of any of these Ca^2+^ handling mechanisms is commonly associated with the development of cardiac dysfunction [10], but whether this is coming from an indirect kidney damage is less known. 

There is a growing body of evidence suggesting that inflammation induced by innate immune system activation can contribute to cardiac dysfunction [11,12]. Indeed, some receptors of the innate immune system are known to play a significant role in the host response after cardiac and renal damage [13,14]. The nucleotide-like receptors (NLRs) are a family of receptors of the innate immune system with a relevant role in several CVDs. Indeed, specific activators of NLRs have a role in the progression of some CVDs [15]. Nucleotide-binding oligomerization domain (NOD) 1 (NOD1) and 2 (NOD2) are members of the NLR family that present a few differences in their stimulatory molecules and tissue location. Regarding their cellular location, NOD1 is broadly expressed in many cell types and organs, such as heart, lung, skeletal muscle and kidney [14,16,17,18], whereas NOD2 expression is more restricted to immune cells and endothelial cells [14,19,20,21,22]. Importantly, different groups including ours have reported an association between NOD1 and CVDs [11,23,24,25,26,27,28,29]. NOD proteins are involved in host defense that respond rapidly to certain pathogens or endogenous molecules released during cell injury by triggering an inflammatory response [17]. NOD1 is a cytosolic protein that contains a caspase activation domain, a recruitment domain (CARD), a nucleotide-binding oligomerization domain (NOD) and a leucine-rich repeat domain. Upon activation, NOD1 undergoes a conformational change, leading to self-oligomerization that allows the recruitment and activation of the receptor-interacting serine/threonine protein kinase 2 (RIP2) through CARD-CARD interactions [30]. Activated RIP2 in turn mediates the recruitment and activation of several mediators that allow the translocation of nuclear factor κB (NF-κB) into the nucleus to initiate the inflammatory response [18].

Several groups, including ours, have analyzed the role of NOD1 in CVD [11,23,31,32,33,34,35]. We previously showed that NOD1 is up-regulated in both mouse and human failing myocardium and its genetic deletion or pharmacological blockade in mice with experimental HF impedes the development of cardiac dysfunction, mainly by preventing cardiac Ca^2+^ mishandling [11]. NOD1 expression has been reported in human and mouse renal tubular epithelial cells [14,36]. Supporting the involvement of NOD proteins in renal disease, Shigeoka et al. demonstrated that the deficiency of *Nod1/2* or *Rip2* deletion, was protective against acute kidney ischemia/reperfusion injury, suggesting that NODs respond to endogenous ligands after injury [14]. By contrast, Stroo et al. reported that the double *Nod1/2* deletion had no impact on the chronic renal damage induced by ureteral obstruction [37].

The potential role of NOD1 in the cardiovascular complications caused by specific renal disease is unknown. Accordingly, the main goal of the present study was to investigate whether the NOD1-dependent pathway was implicated in cardiac dysfunction and Ca^2+^ mishandling induced by experimental CKD.

## 2. Results

### 2.1. Macroscopic and Microscopic Cardiac Features and Biochemical Parameters of Renal Function in Wild-Type and Nod1^−/−^ Mice at Baseline and after Experimental CKD 

Cardiac macroscopic and microscopic parameters of the mice are summarized in Table 1. Macroscopic analysis revealed that the Nx surgery induced an overall loss in BW (body weight) in wild-type (*Wt*) mice, accompanied by a reduction in kidney weight (KW). Notably, the KW in Nx mice considers the remaining one-third of the left kidney after the surgery, which is hypertrophied in both *Wt* and *Nod1*^−/−^ mice (Table 1). The weight of the partial kidney resulting from the surgery (1/6 of the kidney) was similar to the weight of the complete kidney in sham-operated mice, demonstrating that the Nx remaining kidney is hypertrophied in both Wt and *Nod1*^−/−^ mice. KW from *Wt* and *Nod1*^−/−^-sham-operated mice was similar. All these results were supported by KW/BW data (Table 1). Heart weight (HW) and HW/BW ratio were similar between the different groups (Table 1). Additionally, no differences in cardiomyocyte area were observed between *Wt*-sham, *Wt*-Nx, *Nod1*^−/−^-sham and *Nod1*^−/−^-Nx hearts, indicating that Nx surgery does not induce cardiac hypertrophy in *Wt* or *Nod1*^−/−^ mice. Examination of biochemical parameters related to renal function showed comparable kidney impairment in both groups of mice subjected to Nx, as demonstrated by the significantly higher levels of plasma urea and BUN, compared with sham-operated mice (Table 2). FGF-23 levels were higher in *Wt*-Nx and *Nod1*^−/−^-Nx mice compared to sham animals, although they were only significantly increased in *Wt*-Nx, compared to *Wt*-sham. Phosphate levels were not different between the groups. Biochemical kidney parameters were similar between *Wt*-Nx and *Nod1*^−/−^-Nx mice, indicating that the deficiency of NOD1 does not prevent the renal impairment induced by Nx.

### 2.2. Deficiency of NOD1 Prevents both Systolic Ca^2+^ Release Impairment and the Decrease in SR Ca^2+^ Load Triggered by Experimental CKD

**Figure 1:**
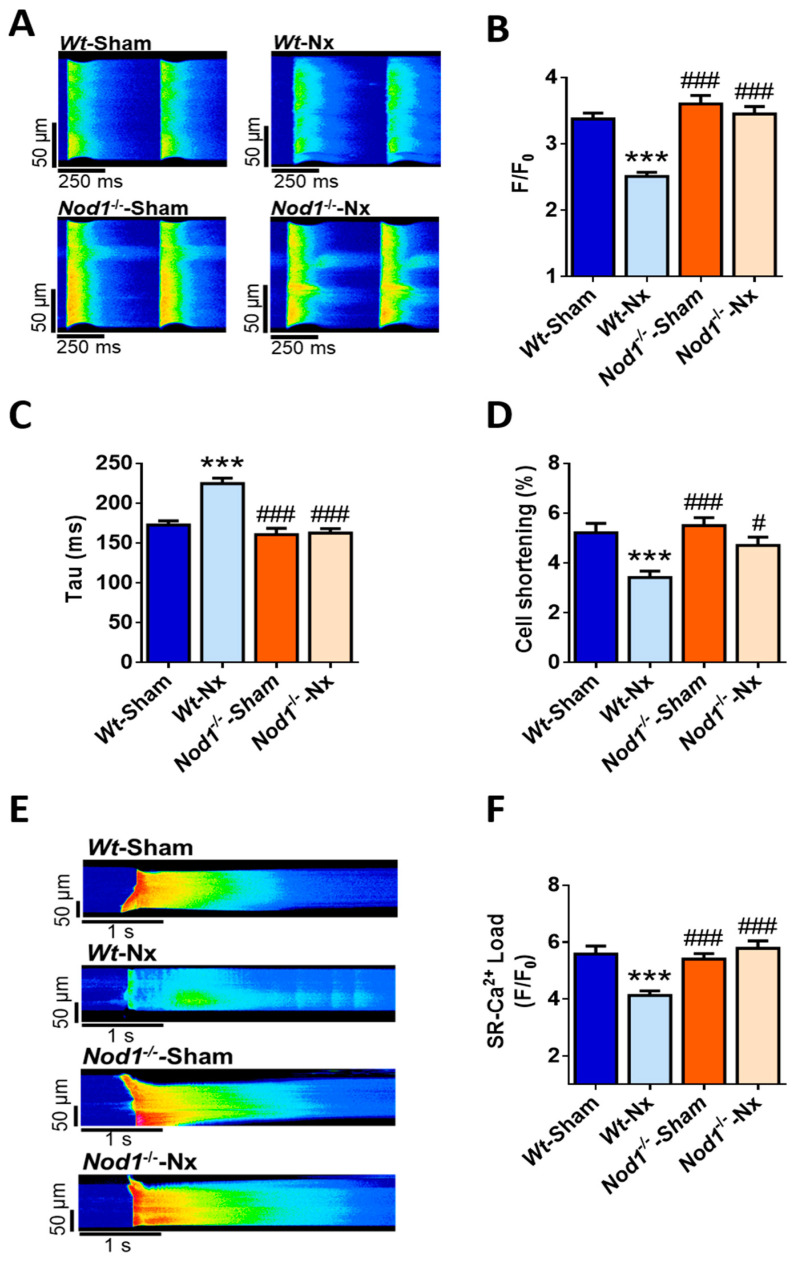
Deficiency of nucleotide-binding oligomerization domain-containing protein 1 (NOD1) prevents the dysregulation of systolic Ca^2+^ release, cell contraction impairment and the reduction in sarcoplasmic reticulum (SR) Ca^2+^-load triggered by 5/6 nephrectomy. (**A**) Representative line-scan confocal images of Ca^2+^ transients in cardiomyocytes from *Wt*-sham, *Wt*-Nx, *Nod1*^−/−^-sham and *Nod1*^−/−^-Nx mice electrically evoked by field stimulation at 2 Hz. Mean values of (**B**) peak fluorescence of Ca^2+^ transients, (**C**) decay time constant and (**D**) cell shortening obtained in cells from *Wt*-sham (*n* = 45 cells/five mice), *Wt*-Nx (*n* = 43 cells/five mice), *Nod1*^−/−^-sham (*n* = 39 cells/five mice) and *Nod1*^−/−^-Nx (*n* = 50 cells/five mice) mice. (**E**) Representative line-scan confocal images of caffeine-evoked Ca^2+^ transients in cardiomyocytes from all groups. (**F**) Mean values of caffeine-evoked Ca^2+^ transients amplitude obtained in cells from *Wt*-sham (*n* = 33 cells/five mice), *Wt*-Nx (*n* = 37 cells/five mice), *Nod1*^−/−^-sham (*n* = 34 cells/5 mice) and *Nod1*^−/−^-Nx (*n* = 35 cells/5 mice) mice. Results show mean ± SEM. *** *p* < 0.001 vs. *Wt*-sham; ^#^ *p* < 0.05, ^###^ *p* < 0.001 vs. *Wt*-Nx.

Given the link between CKD and the prevalence of cardiac and systolic dysfunction [2,3] and the involvement of NOD1 in preventing cardiac Ca^2+^ mishandling [11], we evaluated systolic Ca^2+^ release after Nx and the possible participation of NOD1. To do this, we analyzed cardiomyocyte Ca^2+^ transients electrically-evoked by field stimulation at 2 Hz using confocal microscopy. Representative line-scan images from field-stimulated cardiomyocytes from *Wt*-sham, *Wt*-Nx, *Nod1*^−/−^-sham and *Nod1*^−/−^-Nx mice are shown in Figure 1A. *Wt*-Nx cells clearly displayed a lower amplitude of the intracellular Ca^2+^ transients, slower kinetics and lower cell shortening values than *Wt*-sham cardiomyocytes (Figure 1A–D). By contrast, the amplitude of Ca^2+^ transients in *Nod1*^−/−^-sham and *Nod1*^−/−^-Nx myocytes was very similar, and close to the values in *Wt*-sham cells (Figure 1B). These data indicate that deficiency of NOD1 prevents the decrease in the Ca^2+^ transient’s amplitude, and the impairment in their decay time and cell shortening induced by Nx. Since changes in systolic Ca^2+^ release are closely related to an alteration in the amount of Ca^2+^ that entries though L-type Ca^2+^ channels (LTCCs), we measured the density of I*_CaL_* using patch-clamp technique in the whole cell configuration in cardiomyocytes isolated from *Wt*-sham and *Wt*-Nx mice. Supplementary Figure S1 shows a similar I*_CaL_* density in both experimental groups, indicating that the Nx did no induce any change in the density of Ca^2+^ entering through LTCCs.

We next examined whether the observed differences in Ca^2+^ transients between the different groups were related to changes in the cardiomyocyte SR Ca^2+^ load by measuring caffeine-evoked Ca^2+^ transients. Figure 1E shows representative line-scan images of caffeine-evoked Ca^2+^ transients in each group. The amplitude of caffeine-evoked Ca^2+^ transients was significantly lower in *Wt*-Nx cells than in *Wt*-sham cardiomyocytes (Figure 1E,F). By contrast, this parameter was similar between *Nod1*^−/−^-Nx and *Nod1*^−/−^-sham cardiomyocytes (Figure 1E,F), indicating that NOD1 deficiency prevents the impairment of systolic Ca^2+^ release induced by Nx and contributes to the maintenance of the physiological levels of the SR Ca^2+^ load, allowing adequate systolic Ca^2+^ release and regular cell shortening after experimental CKD.

### 2.3. Deficiency of NOD1 Blunts the Increase in Diastolic Ca^2+^ Release Induced by Nx

**Figure 2:**
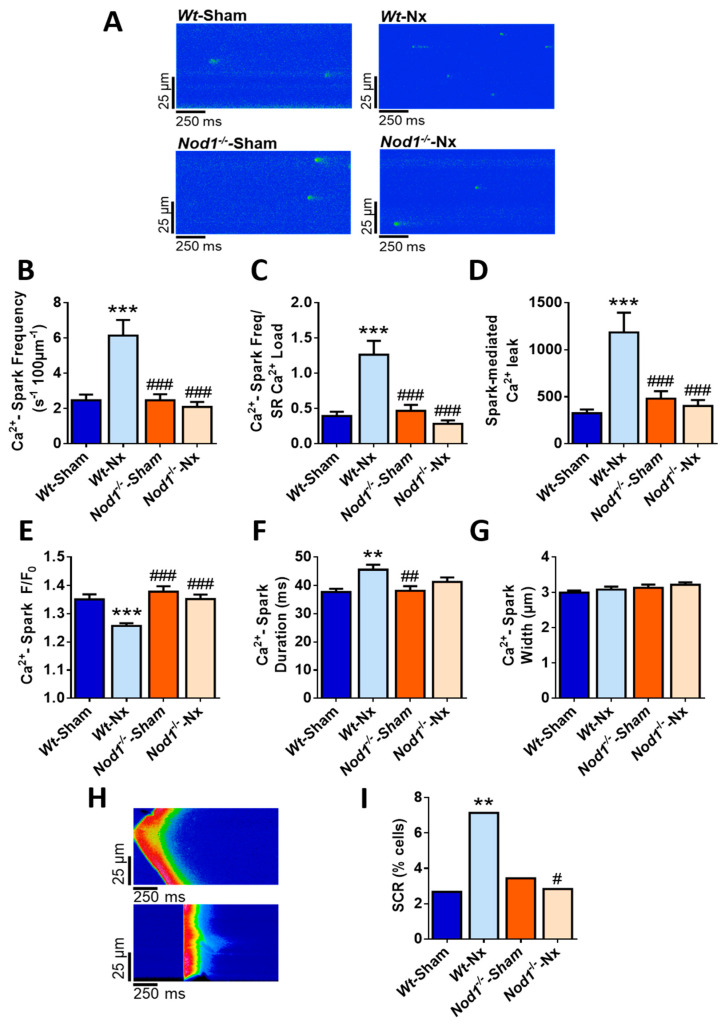
Prevents the increased frequency of Ca^2+^-sparks and spontaneous Ca^2+^ release (SCR) induced by 5/6 nephrectomy. (**A**) Representative line-scan confocal images of Ca^2+^ sparks recordings obtained in a quiescent cardiomyocyte isolated from *Wt*-sham, *Wt*-Nx, *Nod1*^−/−^-sham and *Nod1*^−/−^-Nx mice. Average data of (**B**) Ca^2+^ spark frequency, (**C**) normalization of Ca^2+^ spark frequency by SR-Ca^2+^ load, (**D**) spark-mediated Ca^2+^ leak and Ca^2+^ sparks properties: (**E**) peak, (**F**) duration and (**G**) width obtained in cells isolated from *Wt*-sham (*n* = 45 cells/five mice), *Wt*-Nx (*n* = 47 cells/five mice), *Nod1*^−/−^-sham (*n* = 40 cells/five mice) and *Nod1*^−/−^-Nx (*n* = 46 cells/five mice) mice. (**H**) Representative line-scan confocal images of SCR recordings (Ca^2+^ wave [upper panel]; spontaneous Ca^2+^ transients release (lower panel)) from cardiomyocytes isolated from *Wt*-Nx mice. (**I**) Average data of SCR occurrence obtained in cells isolated from all groups. Histograms show mean ± SEM. ** *p* < 0.01; *** *p* < 0.001 vs. *Wt*-sham; ^#^ *p* < 0.05; ^##^ *p* < 0.01; ^###^ *p* < 0.001 vs. *Wt*-Nx.

Since impairment of SR Ca^2+^ load is frequently associated with alterations in diastolic Ca^2+^ release, we analyzed the frequency and properties of Ca^2+^ sparks to measure the spark-mediated Ca^2+^ leak from RyR_2_ channels. Representative line-scan confocal images of quiescent cardiomyocytes from *Wt*-sham, *Wt*-Nx, *Nod1*^−/−^-sham and *Nod1*^−/−^-Nx mice are shown in Figure 2A. Results showed a significantly higher frequency of Ca^2+^ sparks in *Wt*-Nx cells than in *Wt*-sham counterparts, whereas Ca^2+^ spark frequency in *Nod1*^−/−^-Nx cardiomyocytes was similar to those of in *Nod1*^−/−^-sham cells, and both were comparable with those of *Wt*-sham cells (Figure 2B). Estimation of Ca^2+^ spark frequency normalized to the SR Ca^2+^ load showed that this was significantly higher in *Wt*-Nx cardiomyocytes (Figure 2C). Confirming these data, the overall spark-mediated Ca^2+^ leak was substantially increased in *Wt*-Nx cells (Figure 2D). By contrast, both the Ca^2+^ spark frequency/SR Ca^2+^ load (Figure 2C) and the overall spark-mediated Ca^2+^ leak (Figure 2D) were similar in *Nod1*^−/−^-Nx and *Nod1*^−/−^-sham cardiomyocytes.

Examination of the biophysical characteristics of Ca^2+^ sparks revealed that their amplitude was significantly lower in *Wt*-Nx cardiomyocytes than in *Wt*-sham cells (Figure 2E), whereas the opposite was observed for the average duration of Ca^2+^ sparks (Figure 2F). Ca^2+^ spark amplitude and duration in *Nod1*^−/−^-Nx cells was similar to those of *Nod1*^−/−^-sham cells, and both were comparable with those of the *Wt*-sham group (Figure 2E,F). Conversely, the average width of Ca^2+^ sparks was unchanged between groups (Figure 2G).

We next analyzed other forms of spontaneous Ca^2+^ release (SCR), such as Ca^2+^ waves and spontaneous Ca^2+^ transients, in ventricular quiescent cardiomyocytes. Figure 2H illustrates an example of a Ca^2+^ wave (upper panel) and spontaneous Ca^2+^ transient release (lower panel) from cells isolated from *Wt*-Nx mice. Results showed that the occurrence of SCR was almost 3-fold higher in *Wt*-Nx cardiomyocytes than in *Wt*-sham cells (Figure 2I). By contrast, the occurrence of SCR in *Nod1*^−/−^-Nx cells was significantly lower than in *Wt*-Nx cells, and similar to that obtained in *Nod1*^−/−^-sham and *Wt*-sham myocytes (Figure 2I).

Taken together, these results confirm that the loss of NOD1 prevents the increase in diastolic Ca^2+^ leak induced by Nx, a beneficial effect that can also be related to the maintenance of the SR Ca^2+^ load, as observed in *Nod1*^−/−^-Nx cardiomyocytes. This provides an explanation not only for the improvement in the SR Ca^2+^ load, but also for the better systolic Ca^2+^ release observed in *Nod1*^−/−^-Nx cells relative to the *Wt*-Nx group.

### 2.4. Deficiency of NOD1 Prevents the Increase in the Rate of Pro-Arrhythmogenic Ca^2+^ Events Induced by Nx

**Figure 3:**
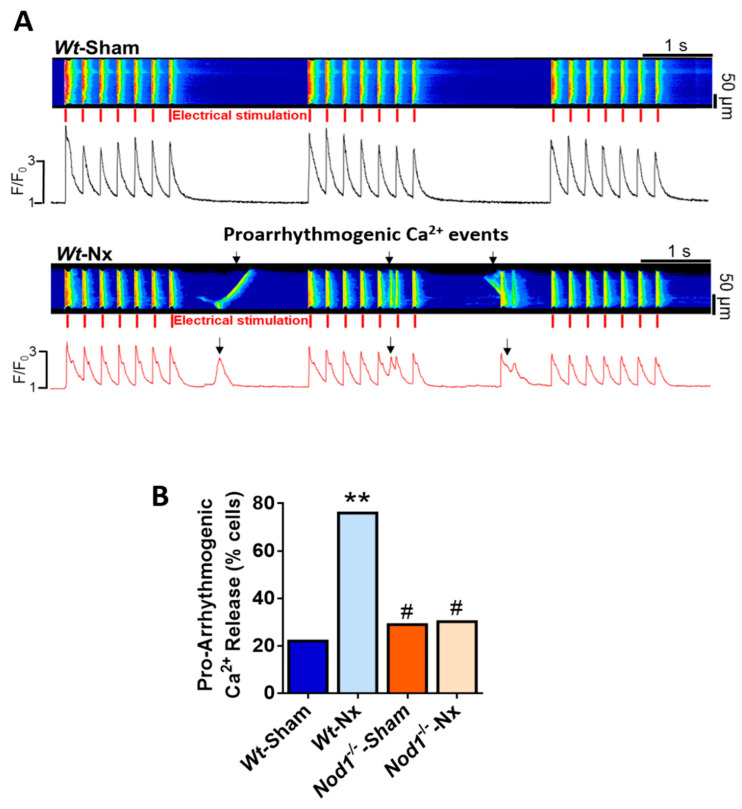
Deficiency of NOD1 prevents the increase of aberrant pro-arrhythmogenic Ca^2+^ events provoked by 5/6 nephrectomy. (**A**) Representative line-scan images of a cardiomyocyte paced at 2 Hz for three cycles isolated from *Wt*-sham (upper panel) and *Wt*-Nx mice (lower panel). The corresponding fluorescence [Ca^2+^]_i_ profiles appear below line-scan images. Red marks indicate electrical stimulation. (**B**) Percentage of cells with pro-arrhythmogenic Ca^2+^ release in cells isolated from *Wt*-sham (*n* = 45 cells/5 mice), *Wt*-Nx (*n* = 47 cells/five mice), *Nod1*^−/−^-sham (*n* = 40 cells/five mice) and *Nod1*^−/−^-Nx (*n* = 46 cells/five mice) mice. Histograms show the mean values. ** *p* < 0.01 vs. *Wt*-sham; ^#^ *p* < 0.05 vs. *Wt*-Nx.

A close relationship exists between altered intracellular Ca^2+^ dynamics and ventricular arrhythmias, which are the most common causes of sudden death in advanced stages of renal disease. We analyzed the occurrence of pro-arrhythmic behavior as spontaneous Ca^2+^ waves or Ca^2+^ transients in ventricular cardiomyocytes field stimulated at 2 Hz for three cycles. Representative line-scan images of a regular Ca^2+^ transient (upper panel) in a *Wt*-sham cell and pro-arrhythmogenic Ca^2+^ transients and waves (lower panel) in a *Wt*-Nx cell are shown in Figure 3A. Results indicated that the occurrence of abnormal Ca^2+^ events was significantly higher in *Wt*-Nx cardiomyocytes than in *Wt*-sham cells, being this pro-arrhythmogenic Ca^2+^ release more than 2-fold higher in *Wt*-Nx (Figure 3B). By contrast, a lower percentage of *Nod1*^−/−^-sham and *Nod1*^−/−^-Nx cells showed this aberrant behavior (Figure 3B). These results indicate that the genetic deletion of NOD1 significantly prevents the increased pro-arrhythmogenic Ca^2+^ release induced by Nx.

### 2.5. Macroscopic and Microscopic Cardiac Features and Biochemistry Parameters of Renal Function of Rip2^−/−^ Mice at Baseline and after Experimental CKD

As the majority of NOD1-derived effects are mediated via RIP2 activation [38] we next analyzed whether the lack of RIP2 also ameliorates Ca^2+^ mishandling linked to CKD.

Similar to the studies in *Nod1*^−/−^ mice, we characterized the model by analyzing the structural properties of both the kidney and heart. Analysis revealed no differences in HW, HW/BW ratio and cardiomyocyte area between *Rip2^−/−^*-sham and *Rip2^−/−^*-Nx mice, indicating that Nx did not induce cardiac hypertrophy (Supplementary Table S1). By contrast, the surgery induced overall BW loss in the *Wt* and *Rip2^−/−^*-Nx mice, along with a reduction in KW in both groups, although the remnant KW after the Nx was higher than one third of the sham-operated mice (Supplementary Table S1). These results were also supported by KW/BW data. Analysis of biochemical indicators of renal function revealed that the levels of plasma urea, BUN and FGF-23 were significantly higher in *Rip2*^−/−^-Nx mice than in *Rip2*^−/−^-sham mice, whereas no differences were observed in phosphate levels between different groups (Supplementary Table S2). The results are similar to those observed in *Wt*-Nx mice, indicating that the loss of RIP2 does not prevent renal impairment induced by Nx.

### 2.6. Deficiency of RIP2 Prevents Ca^2+^ Mishandling Induced by Experimental CKD

**Figure 4:**
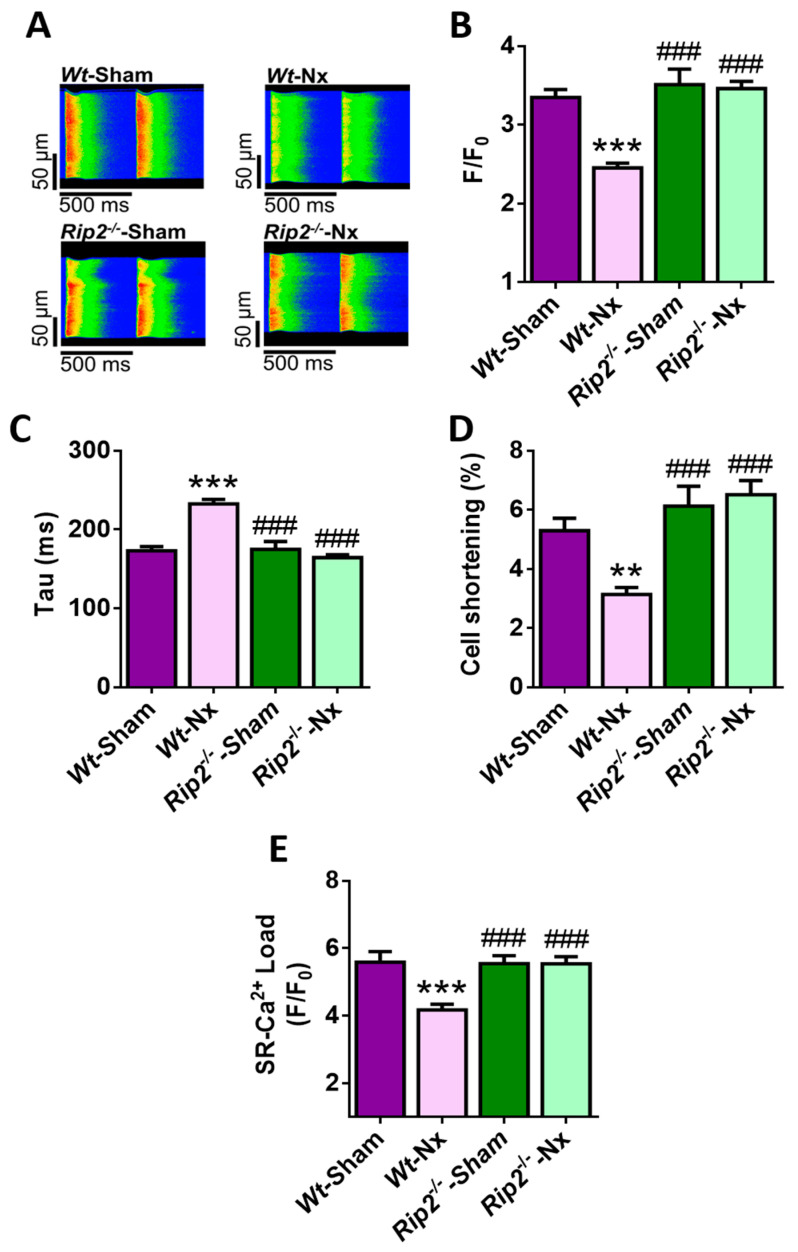
Deficiency of receptor-interacting serine/threonine protein kinase 2 (RIP2) prevents the systolic Ca^2+^ mishandling, contractile dysfunction and depressed SR Ca^2+^-load induced by 5/6 nephrectomy. (**A**) Representative line-scan confocal images of Ca^2+^ transients obtained from *Wt*-sham, *Wt*-Nx, *Rip2^−/−^*-sham and *Rip2*^−/−^-Nx cardiomyocytes electrically evoked under field stimulation at 2 Hz. Mean values of (**B**) peak fluorescence of Ca^2+^ transients; (**C**) decay time constant; and (**D**) cell shortening obtained in cells from *Wt*-sham (*n* = 37 cells/four mice), *Wt*-Nx (*n* = 37 cells/four mice), *Rip2^−/−^*-sham (*n* = 35 cells/four mice) and *Rip2*^−/−^-Nx (*n* = 56 cells/four mice) mice. (**E**) Mean values of caffeine-evoked Ca^2+^ transients amplitude obtained in cardiomyocytes from *Wt*-sham (*n* = 29 cells/four mice), *Wt*-Nx (*n* = 32 cells/four mice), *Rip2^−/−^*-sham (*n* = 24 cells/four mice) and *Rip2*^−/−^-Nx (*n* = 36 cells/four mice) mice. Results show mean ± SEM. ** *p* < 0.01; *** *p* < 0.001 vs. *Wt*-sham; ^###^ *p* < 0.001 vs. *Wt*-Nx.

Analysis of systolic Ca^2+^ release and cell shortening showed that deficiency of RIP2 prevented the lower amplitude of intracellular Ca^2+^ transients (Figure 4A,B), the slower kinetics (Figure 4C) and the decreased cell shortening (Figure 4D) induced by Nx. The changes were also associated with a recovery in the depleted SR Ca^2+^ load levels in *Rip2^−/−^*-Nx cardiomyocytes compared with *Wt*-Nx cells (Figure 4E). Accordingly, cardiomyocytes from *Rip2^−/−^*-Nx mice showed similar systolic Ca^2+^ release and SR Ca^2+^ load, as observed in cells from sham-operated *Rip2^−/−^* and *Wt* mice.

**Figure 5:**
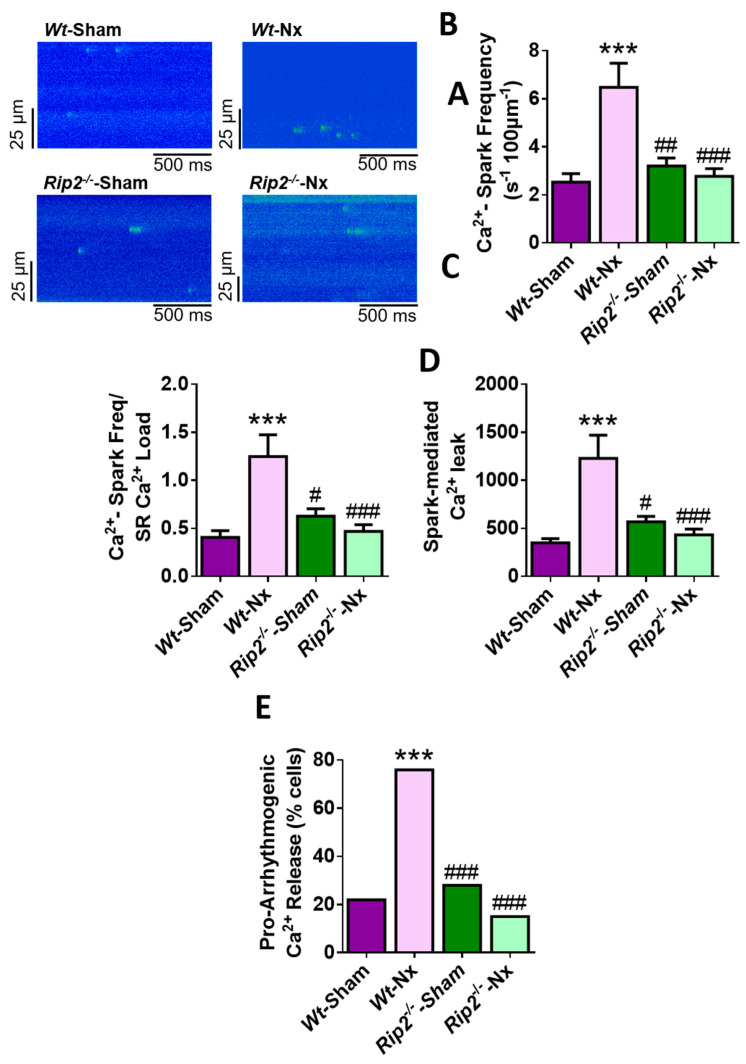
Deficiency of RIP2 prevents the increased frequency of Ca^2+^ sparks, diastolic Ca^2+^ leak and pro-arrhythmogenic Ca^2+^ events provoked by 5/6 nephrectomy. (**A**) Representative line-scan confocal images of Ca^2+^ sparks recordings obtained in a quiescent cardiomyocyte obtained from *Wt*-sham, *Wt*-Nx, *Rip2^−/−^*-sham and *Rip2*^−/−^-Nx. Average data of (**B**) Ca^2+^ spark frequency; (**C**) normalization of Ca^2+^ spark frequency by SR-Ca^2+^ load; (**D**) spark-mediated Ca^2+^ leak in cells isolated from *Wt*-sham (*n* = 35 cells/four mice), *Wt*-Nx (*n* = 38 cells/four mice), *Rip2^−/−^*-sham (*n* = 27 cells/four mice) and *Rip2^−/−^*-Nx (*n* = 47 cells/four mice) mice. (**E**) Percentage of cells with pro-arrhythmogenic Ca^2+^ release in cardiomyocytes isolated all groups. Histograms show mean ± SEM. *** *p* < 0.001 vs. *Wt*-sham; ^#^ *p* < 0.05; ^##^ *p* < 0.01; ^###^ *p* < 0.001 vs. *Wt*-Nx.

Deficiency of RIP2 also prevented the increased diastolic Ca^2+^ leak induced by the Nx surgery. Cells from *Rip2^−/−^*-Nx mice showed similar values of Ca^2+^ sparks frequency, Ca^2+^ sparks frequency normalized by SR Ca^2+^ load and spark-mediated leak to sham-operated *Rip2^−/−^* and *Wt* cardiomyocytes (Figure 5A–D). These data indicate that deficiency of RIP2 also prevents the increased Ca^2+^ leak during diastole and this effect can explain the maintenance of the SR Ca^2+^ load and the physiological systolic Ca^2+^ release observed in the *Rip2^−/−^* model of experimental CKD.

Finally, we determined whether the absence of RIP2 could also modulate the incidence of pro-arrhythmogenic Ca^2+^ release in isolated cardiomyocytes. Results established that only a small number of cells from *Rip2^−/−^*-Nx mice showed pro-arrhythmogenic Ca^2+^ events in paced cells, with the percentage of these events significantly lower than that in the *Wt*-Nx group, and similar to that in sham-operated *Rip2^−/−^* and *Wt* cells (Figure 5E). Overall, these results support the data in *Nod1*^−/−^ mice, and point to a key role for the NOD1 adapter RIP2 in the prevention of Ca^2+^ mishandling induced by experimental CKD.

## 3. Discussion

Our study demonstrates that genetic deletion of either *Nod1* or *Rip2* prevents Ca^2+^ mishandling associated with experimental CKD. Much research has focused on determining the interplay between CVD and CKD [39]; however, many questions remain unanswered, especially in relation to the mechanisms involved in the development of cardiac events after renal damage. Among the multiple risk factors that can explain the high prevalence of CVD in CKD are mineral and bone disorders, oxidative stress, accumulation of uremic toxins and an increased inflammatory response. Regarding CKD, serum Pi levels have been considered a classical biomarker of renal severity and dysfunction together with others such as BUN or urea. However, several authors have already demonstrated that an increase in circulating FGF-23 is the earliest alteration observed in CKD patients even before the increase in serum Pi [40]. In fact, our results point out a similar condition in the experimental CKD developed in mice by the 5/6Nx. Our results demonstrated that the genetic deletion of *Nod1* did not affect the increase in FGF-23 plasma levels. Moreover, the acute incubation of *Wt* and *Nod1*^−/−^ cardiomyocytes with FGF-23 induced a similar systolic and diastolic Ca^2+^ mishandling (Supplementary Figure S2), suggesting that probably NOD-1 and the FGF-23 axis are involved in independent pathways that contribute to the regulation of Ca^2+^ handling, at least in our experimental model of CKD. Importantly, patients with advanced CKD with secondary hyperparathyroidism and hypocalcemia harbor arrhythmias and changes in cardiac electrical conduction [41]. However, an unresolved issue is whether the presence of inflammation is linked to a worse prognosis because of the cardiac events in patients with CKD. Interestingly, the use of specific inhibitors targeting proinflammatory mediators contributes to the prevention of some CKD comorbidities, including cardiovascular complications [42,43]. Sustained activation of the innate immune response leads to increased inflammation and frequently results in maladaptive responses that can promote deleterious cardiac remodeling [13]. NOD1 is an innate immune mediator known to be involved in both CKD and CVD [11,14,23,33,34,35,37,44,45]. With respect to renal diseases, Shigeoka et al. demonstrated that the double *Nod1/2* deletion, as well as *Rip2* deletion, is protective against acute kidney damage induced by ischemia/reperfusion in mice [14]. By contrast, in experimental CKD induced by unilateral ureteral obstruction, Stroo et al. found similar renal damage in *Wt* and double *Nod1*^−/−^/*Nod2^−/−^* mice [37]. These conflicting results might be related to the different experimental procedures used to induce either acute or chronic kidney damage.

We show that deficiency of NOD1 prevents cardiac Ca^2+^ mishandling in a mouse model of CKD induced by 5/6 nephrectomy, suggesting a specific protective cardiac role of this receptor independent of renal damage. The classical 5/6 nephrectomy model of CKD reproduces many of the main features found in human CKD [46,47] and we recently showed that this model presents with elevated cardiac Ca^2+^ mishandling, which can explain the cardiac dysfunction that accompanies CKD [48]. Interestingly, the altered pattern of Ca^2+^ cycling in cardiomyocytes in nephrectomised *Wt* mice has important similarities to that found in HF [11,49], where cardiomyocyte contraction is also strongly compromised. The majority of studies provide evidence that failing hearts show a depressed systolic Ca^2+^ release. As expected, and similar to what occurs in HF, our results show that *Wt*-Nx cardiomyocytes present with a significant decrease in the Ca^2+^ transient amplitude together with a significant slower decay time constant, and having a decreased systolic Ca^2+^ release and depressed cell contraction. All these alterations were not associated with changes in mRNA levels of *Nod1* or *Rip2* (Supplementary Figure S3), suggesting that posttranslational modifications or downstream factors derived from the NOD1-pathway activation can be involved in the observed effects.

In contrast to what occurs in *Wt*-Nx cell, Ca^2+^ mishandling is blunted in *Nod1*^−/−^-Nx mice, chiefly by the prevention of three effects: (i) the decrease in the Ca^2+^ transient amplitude; (ii) the increase in their decay time constant; and (iii) the depressed cell contraction. Thus, the loss of NOD1 prevents the decline in systolic Ca^2+^ release induced by the Nx surgery. This improvement in systolic Ca^2+^ release can be related to the levels of SR Ca^2+^ load. Indeed, the reduction in the SR Ca^2+^ load observed in *Wt*-Nx cardiomyocytes was also prevented by the loss of NOD1. Thus, both the maintenance of the SR Ca^2+^ load and the improvement in the systolic Ca^2+^ release can explain the better cardiac parameters exhibited by *Nod1*^−/−^-Nx cells, compared with *Wt*-Nx counterparts.

Depressed SR Ca^2+^ load can result from an increase in the Ca^2+^ leak during diastole. In this regard, *Wt*-Nx cardiomyocytes showed an increase in diastolic Ca^2+^ leak represented by a higher frequency of Ca^2+^ sparks, Ca^2+^ waves and spontaneous Ca^2+^ transients, as compared with *Wt*-sham cells. Supporting these results, we previously showed that RyR_2_ channel activity is increased in hearts from *Wt*-Nx mice [48]. The increased diastolic Ca^2+^ leak observed in *Wt*-Nx mice is potentially a good substrate for the induction of cardiac arrhythmias since the released Ca^2+^ diffuses to neighboring RyR_2_ clusters inducing SCR and triggering cardiac arrhythmias. The genetic deletion of NOD1 reduces the occurrence of Ca^2+^ sparks, Ca^2+^ waves and spontaneous Ca^2+^ transients after Nx surgery, similar to those found in sham-operated *Wt* and *Nod1*^−/−^ mice. Thus, NOD1 deficiency prevents the abnormal diastolic Ca^2+^ leak induced by the Nx, along with a reduction of pro-arrhythmogenic Ca^2+^ events. A likely explanation for this is that NOD1 deficiency rescues the SR Ca^2+^ content and improves cell contractility, recovering the impaired cardiac outcome observed in *Wt*-Nx mice. These features also bear a resemblance to those found in HF, since SCR is ameliorated in *Nod1*^−/−^-sham mice with experimental HF and the deletion of NOD1 prevents RyR_2_ hyperactivity [11].

To comprehensively study the pathway involved in Ca^2+^ cycling-dysregulation evident in our mouse model of CKD, we also determined whether the deficiency of RIP2 plays a role in the regulation of Ca^2+^ dynamics. RIP2 is the adapter kinase that mediates the majority of NOD1 actions. Accordingly, RIP2-deficient cells are hyporesponsive to signaling through NOD proteins and show severely reduced NFκB activation [50]. Although the role of RIP2 in renal diseases remains enigmatic, its expression has been shown to be strongly induced in failing murine and human myocardium [11]. We demonstrate here that loss of RIP2 prevents the CKD-induced Ca^2+^ mishandling, as cardiomyocytes from *Rip2*^−/−^-Nx mice exhibit improved Ca^2+^ transients amplitude, kinetic rates and cell contractility compared with their *Wt*-Nx counterparts. This improvement in systolic Ca^2+^ release can be due to the rescue of the SR Ca^2+^ content found in the absence of RIP2. Moreover, we also demonstrate that RIP2 deficiency prevents the increased diastolic Ca^2+^ release observed in *Wt*-Nx mice, reducing the Ca^2+^ spark frequency and the occurrence of pro-arrhythmogenic events. These results are in line with those from the analysis of *Nod1*^−/−^ mice. It would be of great interest to determine whether NOD1/RIP2 antagonists can also prevent the Ca^2+^ mishandling linked to CKD in future studies.

In conclusion, we establish, for the first time, to our knowledge, that the genetic deletion of two different components of the NOD1 signaling pathway prevents Ca^2+^ mishandling induced by experimental CKD. Our findings suggest that the NOD1 proinflammatory pathway could be targeted for the development of new therapies to reduce the risk of cardiovascular complications in patients with CKD.

## 4. Methods

### 4.1. Animal Care

The study was conducted following recommendations of the Spanish Animal Care and Use Committee, according to the guidelines for ethical care of experimental animals of the European Union (2010/63/EU), and was approved by the General Direction of Agriculture and the Environment at the Environment Council of Madrid (PROEX: 053/16 and 272.5/20). Male *Nod1*^−/−^ and *Rip2^−/−^* mice on a C57BL/6J (6B; 129P2-NOD^1tm1Nnz^/J: 6B; 129P2-RIP2tm1Nnz/J) background were used. Mice were bred and housed under specific pathogen-free conditions in the Experimental Animal Centre of Instituto de Investigación Hospital Universitario la PAZ, IdiPAZ. Mice were maintained at controlled temperature (23–25 °C) on a 12-hr light/dark cycle with ad libitum access to water and a standard diet. Wild-type (*Wt*) C57BL/6J mice (The Jackson Laboratory, Bar Harbor, ME, USA) were employed as controls. *Nod1*^−/−^ and *Rip2^−/−^* were kindly provided by Dr. Gabriel Nuñez (Ann Arbor, MI, USA). Gene expression analysis of *Nod1* and *Rip2* confirmed the absence of signal in *Nod1-* and *Rip2*-deficient mice (not shown). Macroscopic cardiac and kidney parameters were analyzed in all experimental groups as before [35,49].

### 4.2. Serology

Blood plasma samples were used to analyze the levels of phosphate (Abcam, Cambridge, UK), blood urea nitrogen (BUN) and urea (BioAssays System, Hayword, CA, USA) and fibroblast growth factor-23 (FGF-23) (Immunotopics, Inc., San Clemente, CA, USA) following the manufacturers’ instructions.

### 4.3. Experimental CKD

Six-week-old male were randomly assigned to either five-sixth nephrectomy (Nx) or sham surgery under isoflurane (1.5% *v/v*, isoflurane/oxygen) anesthesia and preoperative analgesia (Metacam, 0.05 mg/kg intramuscular) in a two-stage approach, as described [48]. Briefly, in the first stage an abdominal midline incision was made and the left kidney was exposed. Both the upper and lower poles were tied with a polyglycolic acid suture line (Dexon^®^, 4-0), which was subsequently removed. After a recovery period of one week, the entire right kidney was removed, following ligation of the renal blood vessels and cauterization of the ureter. The peritoneum and skin were then sutured, and the animals were returned to their cages. In control mice, sham surgeries involved midline incision, exposure of both kidneys, but no removal of tissue. The same timings were used as for Nx surgery. Blood plasma was employed for biochemical assays and isolated ventricular cardiomyocytes for Ca^2+^ recordings.

### 4.4. Cardiomyocyte Isolation

Six weeks after the second surgery, ventricular cardiomyocytes were isolated using standard enzymatic digestion [51]. Briefly, mice were anesthetized with sodium pentobarbital (100 mg/kg intraperitoneal) and heparinized (4 U/g intraperitoneal). The heart was rapidly excised and cannulated via the ascending aorta on a Langendorff perfusion apparatus. Retrograde perfusion was initiated with a standard Ca^2+^-free Tyrode’s solution containing 0.2 mmol/L EGTA over 2–3 min at room temperature, and continued for ~3–5 min with the same solution containing collagenase type II (1 mg/mL) (Worthington Biochemical, Lakewood, NY, USA) and CaCl_2_ (0.1 mmol/L). The heart was then removed from the Langendorff apparatus and the ventricles were cut out, finely minced into small pieces and mechanically dissociated in the enzymatic solution (standard Tyrode’s solution containing 0.1 mmol/L CaCl_2_). The cardiomyocyte cell suspension was filtered through a nylon mesh (250 µm), pelleted by centrifugation for 3 min at 300 rpm and suspended in Tyrode’s solution containing 0.5 mmol/L CaCl_2_. Cells were centrifuged as before and suspended in a storage solution containing 1 mmol/L CaCl_2_. Tyrode’s solution comprised (in mmol/L): 130 NaCl, 5.4 KCl, 0.5 MgCl_2_, 25 HEPES, 0.4 NaH_2_PO_4_, 22 glucose; pH = 7.4 adjusted with NaOH. Cardiomyocytes were immediately used for calcium imaging analyses and patch-clamp experiments.

### 4.5. Intracellular Calcium Imaging

Experiments were performed at room temperature (20–23 °C). Images were obtained with a Zeiss LSM 710 Meta confocal microscope (Carl Zeiss, Germany; 40× oil immersion objective with a 1.2 NA), by scanning the cardiomyocytes with an Argon laser every 1 s. Experiments were performed at room temperature (20–23 °C). To record intracellular Ca^2+^ transients, cells were first loaded with the Ca^2+^-sensitive probe Fluo-3 (5 µmol/L; Invitrogen Life Technologies, Carslbad, CA, USA), and were then electrically excited at 2 Hz by field stimulation using two parallel platinum electrodes. Fluo-3 was excited at 488 nm and emitted fluorescence was collected at >505 nm. The fluorescence values (F) were normalized by the basal fluorescence (F_0_) to obtain the fluorescence ratio (F/F_0_). All Ca^2+^ images were corrected for the background fluorescence. The decay time constant of Ca^2+^ transients (Tau) was obtained by fitting the decay trace. Cell contraction was calculated as the difference of cardiomyocyte length between rest and contraction (during electrical stimulation) and expressed as a percentage of shortening of cell length. Spontaneous Ca^2+^ sparks, and spontaneous Ca^2+^ transients and waves were acquired once stimulation was stopped. Ca^2+^ sparks were considered as located and fast increments in Ca^2+^ fluorescence. Total spark-mediated Ca^2+^ leak was calculated by multiplying spark frequency × peak × duration × width. SR Ca^2+^ load was assessed by rapid caffeine (10 mmol/L) application to deplete the SR of Ca^2+^ stores, after field-stimulation to reach the steady-state. Arrhythmic activity was analyzed as abnormal spontaneous Ca^2+^ release (SCR) by applying 3 cycles of field electrical stimulation at 2 Hz paced, consisting of 7 electric pulses. Data analysis was performed with homemade routines using IDL 8 software (Research System Inc. Boulder, CO, USA) and Image J 1.50i software (NIH). Images were corrected for background fluorescence. Cardiomyocyte surface area was quantified with the LSM Zeiss Image Browser 4.2 software (Carl Zeiss).

### 4.6. Statistical Analysis

Results are reported as mean ± SEM. Statistical analysis was performed using one-way analysis of variance (ANOVA) or the chi-square test, as appropriate. If a significant level of *p* was reached (*p* < 0.05) and there was no significant variance in homogeneity, Tukey’s post hoc multicomparison analysis was applied. All statistical analyses were performed with the SPSS 15.0 software (SPSS Inc., Chicago, IL, USA) and significance was assumed when *p* < 0.05.

## Figures and Tables

**Table 1:** Macroscopic parameters in *Wild-type* (*Wt*) and *Nod1*^−/−^ mice subjected or not to experimental chronic kidney disease (CKD).

	*Wt*-sham	*Wt*-Nx	*Nod1*^−/−^-sham	*Nod1*^−/−^-Nx
HW (mg)	186.89 ± 6.08 (10)	169.63 ± 4.65 (8)	198.27 ± 6.66 ^#^ (10)	185.03 ± 12.05 (9)
BW (g)	26.24 ± 0.25 (10)	23.11 ± 0.83 (8)	26.99 ± 0.76 ^##^ (10)	25.26 ± 0.76 (9)
HW/BW (mg/g)	7.11 ± 0.19 (10)	7.43 ± 0.42 (8)	7.38 ± 0.28 (10)	7.31 ± 0.37 (9)
KW (mg)	183.80 ± 4.78 (10)	155.83 ± 8.62 * (8)	190.96 ± 7.02 ^##^ (10)	165.90 ± 7.39 (9)
KW/BW (mg/g)	7.01 ± 0.18 (10)	6.76 ± 0.33 (8)	7.09 ± 0.24 (10)	6.58 ± 0.26 (9)
Cell area (μm^2^)	3482.21 ± 109.25 (72 cells/10)	3215.90 ± 119.73 (54 cells/8)	3396.71 ± 94.36 (67 cells/10)	3271.32 ± 116.62 (59 cells/9)

Data from 8–10 animals for macroscopic parameters per experimental group are reported as mean ± SEM. HW: heart weight, BW: body weight, KW: kidney weight. * *p* < 0.05 vs. *Wt*-sham; ^#^ *p* < 0.05, ^##^ *p* < 0.01 vs. *Wt*-Nx. Statistical significance was determined by one-way analysis of variance (ANOVA).

**Table 2:** Biochemical plasma parameters in *Wild-type* and *Nod1*^−/−^ mice subjected or not to experimental CKD.

	*Wt*-sham	*Wt*-Nx	*Nod1*^−/−^-sham	*Nod1*^−/−^-Nx
Urea (mg/dL)	37.24 ± 3.55 (5)	83.30 ± 5.45 *** (8)	39.80 ± 4.78 ^###^ (6)	83.68 ± 8.04 ***^,^^&&&^ (8)
BUN (mg/dL)	17.40 ± 1.66 (5)	38.93 ± 2.55 *** (8)	18.60 ± 2.23 ^###^ (6)	39.10 ± 3.76 ***^,^^&&&^ (8)
P_i_ (mg/dL)	6.19 ± 0.65 (7)	6.91 ± 0.55 (8)	6.21 ± 0.90 (6)	6.92 ± 1.01 (8)
FGF-23 (pg/mL)	140.3 ± 22.72 (7)	294.00 ± 47.47 * (8)	137.30 ± 24.66 (5)	256.90 ± 38.51 (8)

Data from 5–8 animals for biochemical parameters per experimental group are reported as mean ± SEM. BUN: blood urea nitrogen; FGF-23: fibroblast growth factor 23; Pi: phosphates. * *p* < 0.05, *** *p* < 0.001 vs. *Wt*-sham; ^###^ *p* < 0.001 vs. *Wt*-Nx; ^&&&^ *p* < 0.001 vs. *Nod1*^−/−^-sham. Statistical significance was determined by one-way ANOVA.

## Supplementary Materials

The following are available online at https://www.mdpi.com/1422-0067/21/22/8868/s1.

## Abbreviations

BW	body weight
CARD	caspase activation and recruitment domain
CKD	chronic kidney disease
CVD	cardiovascular disease
EC	excitation-contraction
FGF23	fibroblast growth factor-23
HF	heart failure
HW	heart weight
KW	kidney weight
LTCCs	sarcolemma L-type Ca^2+^ channels
NCX	Na^+^/Ca^2+^ exchanger
NFκB	nuclear factor kappa B
NOD1	nucleotide-binding oligomerization domain-containing protein 1
NOD2	nucleotide-binding oligomerization domain-containing protein 2
Nx	five-sixth nephrectomy
RIP2	receptor-interacting-serine/threonine-protein kinase 2
RyR_2_	ryanodine receptor type 2
SCR	spontaneous Ca^2+^ release
SERCA2a	sarco/endoplasmic reticulum Ca^2+^ pump subtype
SR	sarcoplasmic reticulum
TL	tibia length
Wt	wild-type
