# Supplementary material for: Genetic Deletion of NOD1 Prevents Cardiac Ca2+ Mishandling Induced by Experimental Chronic Kidney Disease
Source: Int J Mol Sci. 2020 Nov 23;21(22):8868. doi: 10.3390/ijms21228868 (PMC7700567; doi:10.3390/ijms21228868)
Supplement: Supplementary file 1 [file ijms-21-08868-s001.pdf]

Supplementary Methodology

Patch-clamp I_{CaL} recordings

I_{CaL} was recorded by the whole-cell voltage-clamp technique using an Axopatch 200B amplifier (Molecular Devices, CA, USA). The patch pipette resistance was 1.0-2 MΩ. Patch clamp experiments were carried out at room temperature. Current traces were digitized with Digidata 1440A and analyzed using pClamp10 software (Molecular Devices, CA, USA). Current density was obtained from the normalization of the current amplitude to the membrane capacitance (Cm). I_{CaL} was obtained by the application of 300 ms depolarizing voltage pulses from the holding potential of -50 mV, from -40 mV to +60 mV at a frequency of 0.2 Hz. The extracellular solution contained (in mM): 140 NaCl, 1.1 $MgCl_2$, 5.4 CsCl, 10 glucose, 5 HEPES, 1.8 $CaCl_2$; pH was adjusted to 7.4 with CsOH. The intracellular recording pipette solution for whole-cell experiments contained (in mM): 100 CsCl, 20 TEACl, 5 EGTA, 10 HEPES, 5 Na_2ATP, 0.4 Na_2GTP, 5 Na_2 creatine phosphate, 0.06 $CaCl_2$; pH was adjusted to 7.4 with CsOH.

RNA isolation and Quantitative Real-Time PCR

For RT-qPCR analyses, total RNA was isolated from the frozen heart tissue using the RNeasy Mini Kit (Qiagen, Hilden, Germany) following the manufacturer's instructions, and quality was assessed with NanoDrop One Microvolume UV-Vis Spectrophotometer (Thermo Fischer Scientific, Waltham, MA). A total of 1 μg of total RNA was reverse-transcribed to cDNA using High-Capacity cDNA Reverse Transcription Kit (Applied Biosystems, Foster City, CA) and RT-qPCR was performed using FastStart Essential DNA Green Master (Roche) in 10 μl total reaction volume on a LightCycler® 480 II (Roche, Basel, Switzerland) at optimized thermocycling settings. mRNA levels for genes of interest were normalized to the mRNA levels ribosomal *36b4* (RPLP0) as housekeeping gene, evaluated using the$2^{-\quad Ct}$ method and expressed as the relative change compared to the control samples. The primer sequences (5′-3′) for NOD1 and

RIP2 were as follows: NOD1-Forward: CCTTGCTTTAGCCGTCTCAC NOD1-Reverse: TCCTCACATAGCACCTTCACC; RIP2-Forward: ATGCCACCTGAGAACTATGAGC; RIP2-Reverse: GCAAAGGATTGGTGACCTCTT; 36b4-Forward AGATGCAGCAGATCCGCAT; 36b4-Reverse GTTCTTGCCCATCAGCACC.

Supplementary Figures

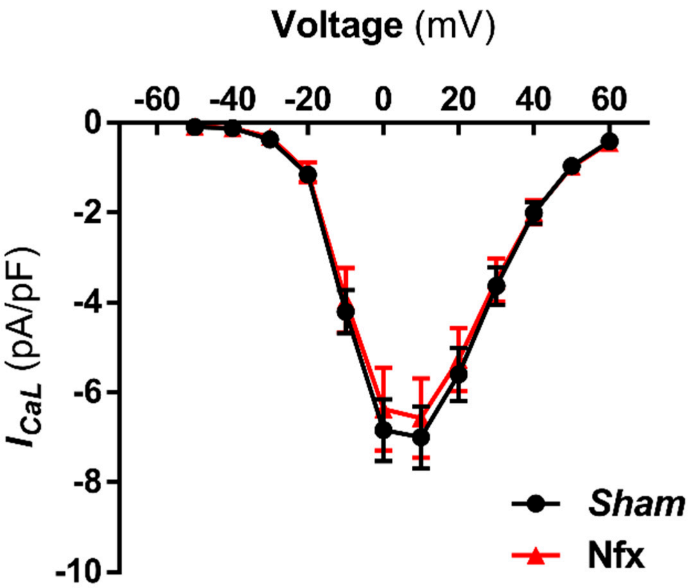

***Supplementary Figure S1*: Density of I_{CaL} was similar in Wt and wt-NFX.** Current density-voltage relationship from *Sham* (n=10 cells/N=3 mice) and Nfx (n=12 cells/N=3 mice) cardiomyocytes. Data are presented as mean±SEM.

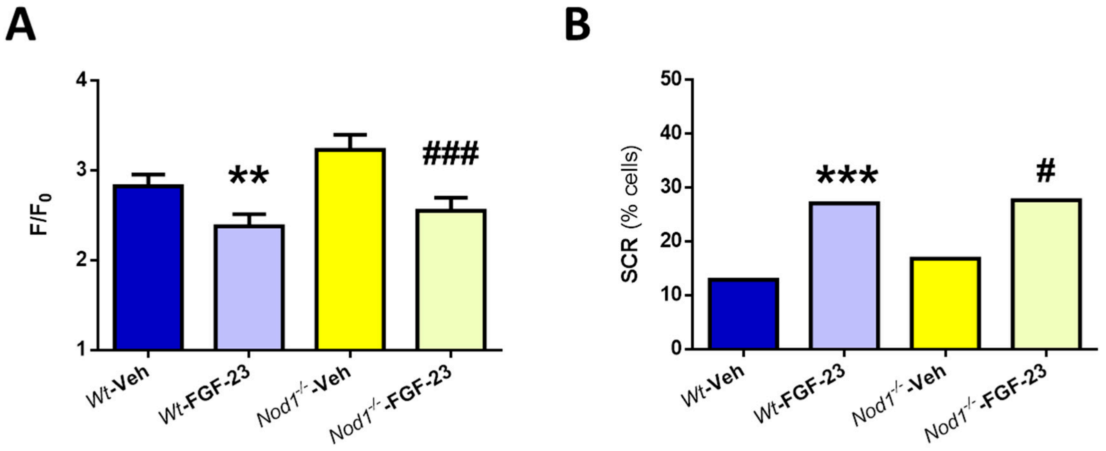

***Supplementary Figure S2*: FGF23 induces similar systolic and diastolic Ca^{2+} mishandling in isolated cardiomyocytes from *Wt* and *Nod1$^{-/-}$* mice.** Amplitude of Ca^{2+} transients (F/F_0, panel A)

and spontaneous Ca^{2+} release (SCR, panel B) obtained in cardiomyocytes isolated from Wt (n=15) or Nod1$^{-/-}$ (n=19) mice and incubated with vehicle or 100 ng/mL FGF-23 for 5 min. Data are presented as mean±SEM.**p<0.01, ***p<0.001 *vs. Wt*; $^{\#}$p<0.05, $^{\#\#\#}$p<0.001*vs. Nod1*$^{-/-}$.

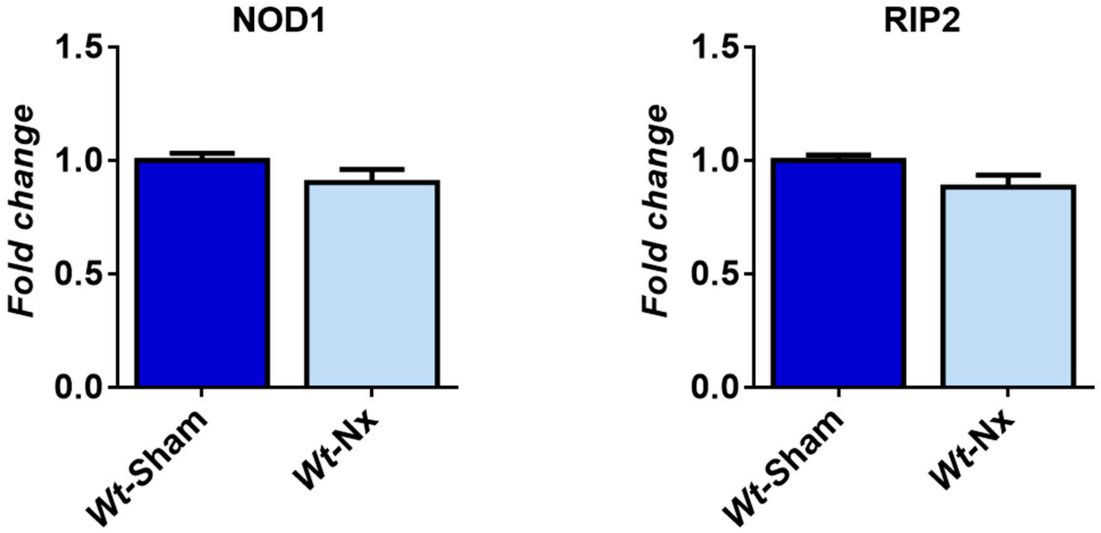

***Supplementary Figure S3*: Cardiac mRNA levels of NOD1 and RIP2 are similar in *Wt*-Sham (N=5) and *Wt*-Nx mice (N=4).** Data are presented as fold change related to 18sRNA and represent mean ± SEM.

Supplementary tables.

Table S1. Macroscopic parameters in *Wild-type* and *Rip2*$^{-/-}$ mice in absence and presence of experimental CKD.

	***Wt*-Sham**	***Wt*-Nx**	***Rip2*$^{-/-}$-Sham**	***Rip2*$^{-/-}$-Nx**
**HW (mg)**	185.10 ± 6.50 (9)	170.50 ± 4.33 (8)	168.86 ± 7.96 (5)	170.63 ± 15.62 (6)
**BW (g)**	26.16 ± 0.26 (9)	23.05 ± 0.83** (8)	24.31 ± 0.58 (5)	22.34 ± 0.91** (6)
**HW/BW (mg/g)**	7.07 ± 0.21 (9)	7.49 ± 0.41 (8)	6.95 ± 0.32 (5)	7.58 ± 0.48 (6)
**KW (mg)**	183.3 ± 5.32 (9)	151.5 ± 10.76* (8)	175.5 ± 7.51 (5)	139.8 ± 6.55$^{**,\&}$ (6)
**KW/BW**	7.01 ± 0.20 (9)	6.59 ± 0.43 (8)	7.22 ± 0.23 (5)	6.29 ± 0.32 (6)

**(mg/g)**				
**Cell area (μm^2)**	3565.11 ± 118.6 (61 cells/9)	3274.45 ± 128.0 (46 cells/8)	3198.86 ± 87.64 (85 cells/5)	3171.50 ± 110.0 (60 cells/6)

Data from 5-9 animals for macroscopic parameters per experimental group are reported as mean ± SEM. **HW**: heart weight, **BW**: body weight, **KW**: kidney weight. *p <0.05, **p <0.01 vs. *Wt*-Sham; $^{\&}P$<0.05 vs. *Rip2*$^{-/-}$-Sham. The p-values were determined using one-way ANOVA test.

Table S2. Biochemical plasma parameters in *Rip2*$^{-/-}$ mice in absence and presence of experimental CKD

	***Rip2*$^{-/-}$-Sham**	***Rip2*$^{-/-}$-Nx**
**Urea (mg/dL)**	46.06 ± 3.06 (5)	104.84 ± 10.25*** (6)
**BUN (mg/dL)**	21.52 ± 1.43 (5)	48.99 ± 4.79*** (6)
**P_i (mg/dL)**	9.78 ± 1.19 (5)	10.01 ± 0.64 (6)
**FGF-23 (pg/mL)**	234.60 ± 31.75 (5)	450.70 ± 55.69* (6)

Data from 5-6 animals for biochemical parameters per experimental group are reported as mean ± SEM. **BUN**: blood urea nitrogen; **FGF-23**: fibroblast growth factor 23; **Pi**: phosphates. *P<0.05, ***P<0.001. The p-values were determined using Student's t-test.
